# Etched Tungsten Oxide Modified with Au for Quick Xylene Detection

**DOI:** 10.3390/mi16060646

**Published:** 2025-05-28

**Authors:** Yinglin Wang, Zhaohui Lei, Xu Li, Yantong Meng, Wanting Cui, Yiyang Xu, Xidong Hao, Shanfu Sun, Pengfei Cheng

**Affiliations:** School of Aerospace Science and Technology, Xidian University, Xi’an 710126, China; lzhcatgod@163.com (Z.L.); xulee1014@foxmail.com (X.L.); mytxxxd@163.com (Y.M.); cuiwanting0719@163.com (W.C.); xyy13243996167@163.com (Y.X.); haoxidong@xidian.edu.cn (X.H.); sunshanfu@xidian.edu.cn (S.S.)

**Keywords:** WO_3_, gas sensor, xylene sensor, Au decoration, etching techniques

## Abstract

Due to its widespread distribution in industrial, commercial, and residential settings, xylene detection is crucial. In this study, carbon sphere templates and NaHCO_3_ etching were used to synergistically prepare WO_3_ with uniform macropores, which was then decorated with Au elements. The findings demonstrated that the Au-decorated WO_3_-etched sample (WO_3_-1%E+Au) had the best sensing performance for 100 ppm xylene (response value: 21.3, optimal operating temperature: 360 °C) and short response/recovery time (1 s/11 s). The etching of NaHCO_3_ and the synergistic carbon sphere templates were responsible for the sensing performance, as they enhanced the sample surface’s specific surface area and roughness while also supplying additional active sites. Furthermore, the sensor’s sensitivity and selectivity to xylene were enhanced by the coupling effect and dehydrogenation catalysis of the noble metal Au. The results of this work advance our knowledge of gas-sensing mechanisms and offer guidance for the creation of extremely sensitive and selective xylene gas sensors.

## 1. Introduction

Xylene is a volatile organic compound (VOC) of great concern that is employed in a wide variety of industrial, commercial, and household applications. It is commonly found in paints, solvents, adhesives, and cleaners [1,2,3]. On the other hand, exposure to xylene vapors can have harmful impacts on human health, including respiratory irritation, dizziness, and even chronic issues [4,5]. Furthermore, xylene is regarded as a possible environmental toxin with the potential to contaminate air, soil, and water [6]. Therefore, it is crucial to create an effective and dependable gas sensor for xylene detection and monitoring in order to preserve the environment, public health, and workplace safety [5,6].

Due to their high response values and high carrier mobility for gas detection, n-type metal oxide semiconductors have been used extensively among MOS [7,8,9]. For gas sensors, tungsten oxide (WO_3_) has shown great promise as an n-type MOS material because of its excellent physical and chemical properties [10,11]. In order to further enhance the performance of gas sensors (e.g., increase the sensitivity, improve the selectivity, and enhance the speed of response and recovery), researchers usually carry out studies in the following aspects, including increasing active surface sites and second element-induced catalysis [12,13].

Etching or surface modification has been thought to be a successful strategy for improving gas sensor performance. By increasing the sensing materials’ surface area, surface pore density, and surface roughness, this technique creates additional active sites for gas molecule interaction [14,15,16,17,18,19]. Radzali utilized photoelectrochemical etching to effectively fabricate porous InAlGaN [20]. Compared to the nonporous InAlGaN, the sensor demonstrated a sensing response to hydrogen that was around 6.9 times higher. The response and recovery times of the sensor were 13.8 s and 88.4 s at room temperature. With the aid of KOH, Qin prepared well-aligned, loosely structured rough silicon nanowire arrays with large surface area using metal-assisted chemical etching [21]. The sensor exhibited a satisfactory response value of 83% to 200 ppm H_2_. KOH treatment roughened the surface to increase the surface area and improve the gas adsorption capacity.

Through chemical and electronic sensitization, the utilization of noble metals like Au [22], Pd [23], Pt [24], and Ag [25] as second elements added to the sensitizer materials may enhance the sensing performance of target gas [26]. In the meantime, noble metals usually have the selectivity modulate function for target gases [27,28]. Kim reported a study that involved loading several noble metal catalysts onto In_2_O_3_ to regulate the selectivity of gas sensors. Ru and Pd catalysts showed excellent selectivity to CH_3_SH and H_2_S, while Pt interacted preferentially with CH_3_COCH_3_ [27]. Both the catalytic affinity of each noble metal for the target gas and the distinct catalytic effects arising from the metal-sensitizer material interactions are responsible for the variation in sensitivity of these three sensors. Some noble metals can also react selectively with particular gases, and in Barbosa’s study, Pd-SnO_2_ showed significantly higher response values than Ag-SnO_2_ and pristine SnO_2_ for varying concentrations of H_2_ under the same detection conditions [28]. This is mostly because Pd adsorbs H_2_ in a particular way, separates it into H atoms, and then combines with the separated H atoms to form PdH_x_ [26].

This study successfully synthesized uniformly macroporous WO_3_ in a water bath with NaHCO_3_ as etchant and carbon spheres as support. For a variety of VOCs, the gas detection capabilities of every sensor were examined. The results demonstrated that the WO_3_-1%E+Au gas sensor exhibits outstanding gas-sensing performance, including high response and short response and recovery time (tres/trec = 1 s/11 s). In addition, a detailed discussion of the improved sensing mechanism via macroporous structure following etching and Au decorating is provided.

## 2. Materials and Methods

The reagents used in the experiment are of analytical grade and do not require further purification. Glucose (C_6_H_12_O_6_), phosphotungstic acid hydrate (12WO_3_∙H_3_PO_4_∙xH_2_O), ethanol (C_2_H_6_O), Sodium bicarbonate (NaHCO_3_) and chloroauric acid (HAuCl_4_H_2_O) were purchased from Sinopharm Co. Ltd, Beijing, China.

### 2.1. Materials Synthesis

The preparation of carbon spheres has been reported in our previous work [29]. A total of 6 g of glucose was dissolved in 50 mL of deionized water and transferred into a 50 mL polytetrafluoroethylene-lined container. This was subsequently placed inside a stainless-steel reactor and heated at 180 °C for 12 h. At the end of the reaction, a black sandy product would be obtained. The product was washed alternately with deionized water and ethanol to wash out impurities. The final product was dried at 80 °C overnight for further experiments. The final carbon sphere template was obtained. The SEM image of carbon spheres can be seen in Figure S1. As depicted, the particle size of carbon spheres was mainly concentrated around 500−600 nm.

In general, 0.886 g phosphotungstic acid hydrate and 0.12 g carbon spheres were dissolved in 50 mL deionized water (DI water) and ultrasonicated for 15 min with constant stirring. The reaction was then carried out in a water bath at 80 °C for 4 h. At the end of the reaction, the precursor was washed six times with DI water and dried at 60 °C. Finally, the precursors were calcinated in air at 550 °C for 2 h at a ramp rate of 2 °C/min. The resulting powder sample was labeled as WO_3_.

The NaHCO_3_-etched samples were prepared as follows: in the same manner as described above for the WO_3_ samples, after adding phosphotungstic acid and carbon spheres to 45.5 mL of deionized water, 4.5 mL of NaHCO_3_ solution (at a concentration of 1 mg/mL) was also added to the above solution. The synthesized sample was labeled as WO_3_-1%E.

The WO_3_-1%E+Au sample was prepared in the same way as the WO_3_-1%E sample, except that 7.6 μL (0.02 g/mL) of chloroauric acid solution (equivalent to 1 mol% of Au) was added to the solution.

### 2.2. Materials Characterization

The X-ray diffraction (XRD) peaks were measured by a Bruker D8 Advance (Bruker, Bremen, Germany) operated at 40 kV, 300 mA, using Cu Kα radiation with the wavelength of 1.5418 Å and a scan angle of 20–80°. The morphology and phase composition of the samples were observed by field emission scanning electron microscopy (FESEM, FEI, Hillsborough, NC, USA) and transmission electron microscopy (TEM, Titan G260-300, FEI, Hillsborough, NC, USA). X-ray photoelectron spectroscopy (XPS, AXISULTRA Karatos, Kyoto, Japan) was obtained with a monochromatic Al Kα (1486.71 eV) line under a vacuum condition of ~10-8 Torr at 100 W power. The specific surface area of nitrogen adsorption-desorption was recorded on a Quantachrome Autosorb-iQ (Guoyi Quantum Co., Ltd., Heifei, China). The pore size and its distribution were calculated by the Barrett-Joyner-Halenda (BJH) method.

### 2.3. Gas Sensor Fabrication and Measurement

The prepared sample was thoroughly mixed with DI water and then ultrasonicated to obtain a slurry. The slurry was carefully applied to the surface of the ceramic tubes with a brush, followed by calcination at 350 °C for 2 h with a heating rate of 1 °C/min. After cooling to room temperature, a heating wire was carefully inserted into the ceramic tube. The ceramic tube and the inserted heating wire were fixed on a six-legged base. To stabilize the electrical properties of the prepared sensors, the assembled devices were aged for 5–7 days.

The devices were evaluated in a static system in a 1 L test chamber under controlled conditions of 25 °C and 30 ± 5% relative humidity. The working temperature of the device was adjusted by an internal heating resistance wire. The output resistance of the device was continuously monitored using a digital resistance meter (Fluke 8846A). The resistance of the sensor stabilized in the air environment, at which point the resistance value was marked as R_a_. A liquid corresponding to the concentration of the test gas was withdrawn using a micro-syringe [30,31], injected into the 1 L test chamber, and mixed homogeneously with air. The sensor was then placed into the test gas chamber. After the resistance of the sensor in the test chamber stabilized, the resistance value was marked as R_g_. Finally, the sensor was placed into a clean air chamber to return the resistance value to its initial state. The response of the gas sensor to the target gas is identified as the ratio (R_a_/R_g_) between the resistance in air (R_a_) and the resistance value in the target gas (R_g_). The response time (tres) and recovery time (trec) of the sensor are determined based on the time required for the sensor to reach 90% of the total change in resistance exposed to the target gases and fresh air, respectively [32].

## 3. Results

### 3.1. Structural and Morphological Characteristics

**Figure 1:**
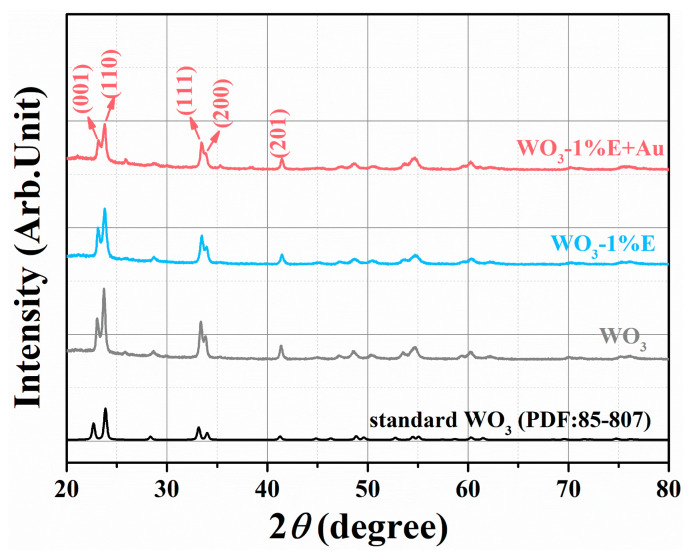
X-ray diffraction patterns of all samples.

The crystalline phases of all the samples were characterized by XRD. In Figure 1, the diffraction peaks of WO_3_ can be categorized as monoclinic WO_3_ (PDF: 85-807) with lattice parameters of a = 7.297 nm, b = 90.91 nm, and c = 7.688 nm. No impurity phases were detected, implying that the material is of high purity. For the WO_3_-1%E+Au sample, there was no diffraction peak corresponding to the Au element due to the low addition of Au (1 mol%). According to the Debye-Scherrer formula, the grain sizes of all samples are approximately 34.73 nm, 24.36 nm, and 26.21 nm [33]:

D=0.9λ/βcos⁡(θ)

Here, λ is the wavelength of X-ray radiation, λ = 0.1542 nm, β is the full width at half maximum (FWHM), and θ is the Bragg diffraction angle. Compared to the WO_3_ sample, WO_3_-1%E and WO_3_-1%E+Au samples exhibit smaller grain sizes. A smaller grain size usually indicates a more compact and ordered crystal structure with higher lattice strain [34,35]. Smaller grain size also produces a larger surface area at the grain boundaries, which provides more active sites and reactivity [36] and effectively promotes charge transfer and surface reaction with the target gas [37].

**Figure 2:**
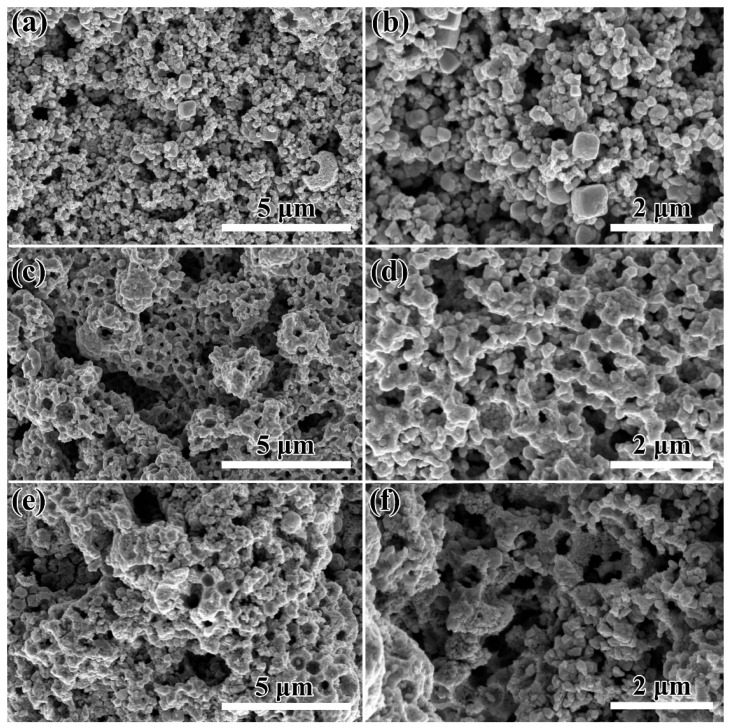
SEM images of (**a**,**b**) WO_3_, (**c**,**d**) WO_3_-1%E, and (**e**,**f**) WO_3_-1%E+Au.

The morphology of the prepared samples was evaluated by FESEM. Figure 2a,b shows the unetched WO_3_ samples. The WO_3_ samples prepared using phosphotungstic acid and carbon spheres produced WO_3_ particles with an average size of about 200−300 nm after calcination. The carbon spheres did not act as supporting templates in the morphology formation of the WO_3_ samples, mainly due to the weak interaction between the carbon sphere templates and phosphotungstic acid, which was not sufficient to affect the growth morphology of the WO_3_ nanoparticles.

WO_3_-1%E samples with uniform macroporous structure were obtained after the addition of etchant NaHCO_3_ to the reaction solution (Figure 2c,d and Figure S2). This may be attributed to the fact that NaHCO_3_, as a strong alkaline and weakly acidic etchant, dissolves and binds the boundaries of independently grown WO_3_ nanoparticles to each other, encapsulating the carbon sphere templates. After removing the carbon spheres by sintering, the porous structured WO_3_ consisting of boundary fuzzy particles was finally obtained (Figure 2d). The SEM images of the WO_3_-1%E+Au samples are shown in Figure 2e,f. The morphology of the samples was not much affected by the introduction of chloroauric acid, and the particle morphology of the samples still maintained a uniform porous structure.

In addition, in order to investigate the effect of NaHCO_3_ content on the product morphology, different concentrations of NaHCO_3_ (0.5% NaHCO_3_, 1% NaHCO_3_, 3% NaHCO_3_, and 5% NaHCO_3_) were selected and added to the reaction system, and the SEM images of the samples obtained are shown in Figure S3. It can be seen that at lower NaHCO_3_ concentrations, the etching effect on WO_3_ particles is not obvious, and the particle boundaries are not effectively bound. With the increase in NaHCO_3_ concentration, the size of WO_3_ particles decreases gradually. It is worth noting that when the concentration of NaHCO_3_ rises, the inter-particle bonding breaks down, causing the carbon spheres to lose their ability to sustain the sensing materials.

**Figure 3:**
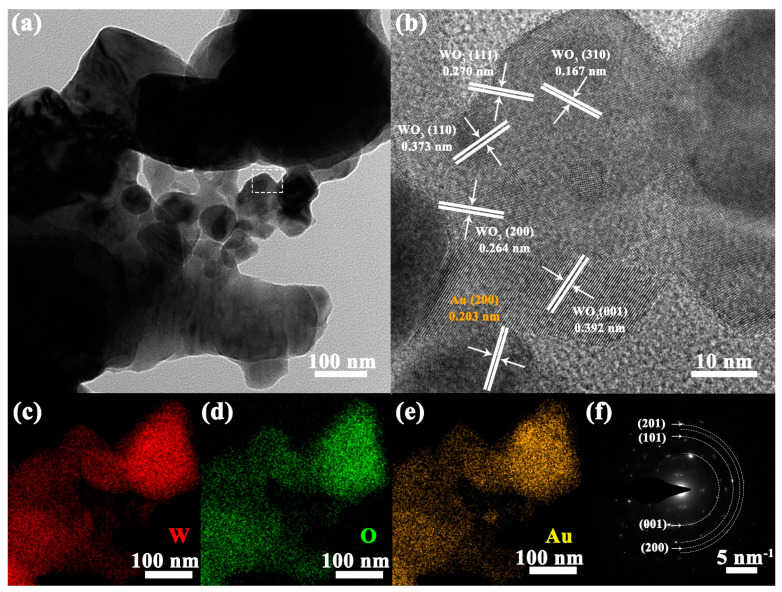
WO_3_-1%E+Au sample: (**a**) TEM image, (**b**) High-resolution TEM selected area image of (**a**), (**c**–**e**) Elemental mapping images of W, O, and Au elements, and (**f**) Selected area electron diffraction image.

TEM and HRTEM measurements were used to analyze the morphology and structure of the WO_3_-1%E+Au sample. As shown in Figure 3a, the sample was formed by the accumulation of particles. Figure 3b shows the HRTEM image within the white dashed box in Figure 3a, where the crystal spacing corresponding to the WO_3_ (111), (310), (110), (200), and (001) crystal planes and the Au (200) crystal plane can be clearly observed. In addition, the EDS (Energy Dispersive X-ray Spectroscopy) maps in Figure 3c–e show the distribution of tungsten (W), oxygen (O), and small amounts of gold (Au) in the WO_3_-1%E+Au sample. It can be seen that the Au element is uniformly distributed in the WO_3_ sensitive matrix. Figure 3f shows a typical pattern of WO_3_ where characteristic monoclinic reflections are observed, corresponding to the (201), (101), (200), and (201) crystal planes of WO_3_.

**Figure 4:**
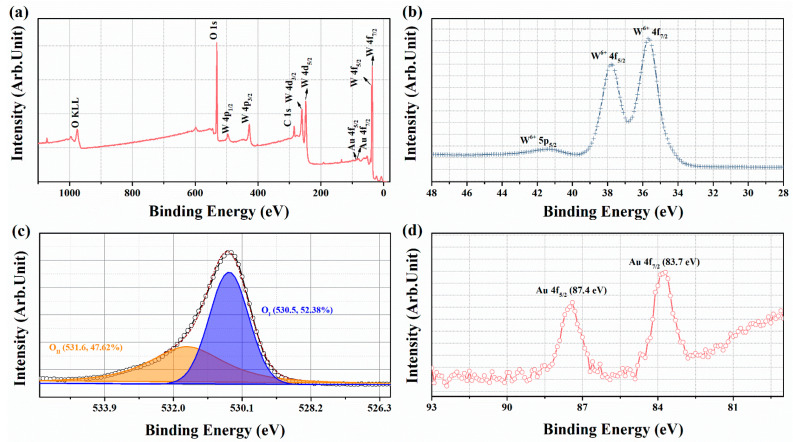
The XPS spectra of WO_3_-1%E+Au sample: (**a**) Full survey spectra, (**b**) W^6+^, (**c**) O 1s, (**d**) Au 4f.

XPS detection was used to determine the chemical valence states of the elements in the samples. For the WO_3_-1%E+Au sample, a wide scan XPS spectrum was obtained between 0 and 1100 eV (Figure 4a), and the peaks were observed matching the elements C, W, O, and Au. The XPS measurements were calibrated using the characteristic carbon peak (C 1s) with a binding energy of 284.8 eV. In the XPS spectrum (Figure 4b), the peaks matching the W element can be observed at binding energies of 41.3 eV, 37.8 eV, and 35.7 eV, corresponding to W^6+^ 5p_5/2_, W^6+^ 4f_5/2_, and W^6+^ 4f_7/2_ [38,39].

In Figure 4c, the O 1s peak of WO_3_-1%E+Au exhibits asymmetry and can be decomposed into two distinct components: lattice oxygen (O_I_) with lower binding energy and surface oxygen (O_II_) with higher binding energy [40,41]. Surface oxygen can be attributed to oxygen species such as O^−^ O^2−^, or OH^−^ on the WO_3_ surface [42]. These oxygen species play a significant role in improving the gas-sensing performance. The O 1s spectra of WO_3_ and WO_3_-1%E are shown in Figure S4. The calculated content of OII in WO_3_-1%E+Au (47.62%) is higher than that of WO_3_ (46.04%) and WO_3_-1%E (46.76%). This suggests that the Au loading on the surface of WO_3_ enhances the adsorption of the oxygen species, thus providing more opportunities for the absorption of the detected gas. Figure 4d depicts the Au 4f peaks for WO_3_-1%E+Au. The peaks observed at 83.7 eV and 87.4 eV correspond to Au 4f_7/2_ and Au 4f_5/2_, respectively, which can predominantly be ascribed to the presence of metallic Au^0^ [43].

**Figure 5:**
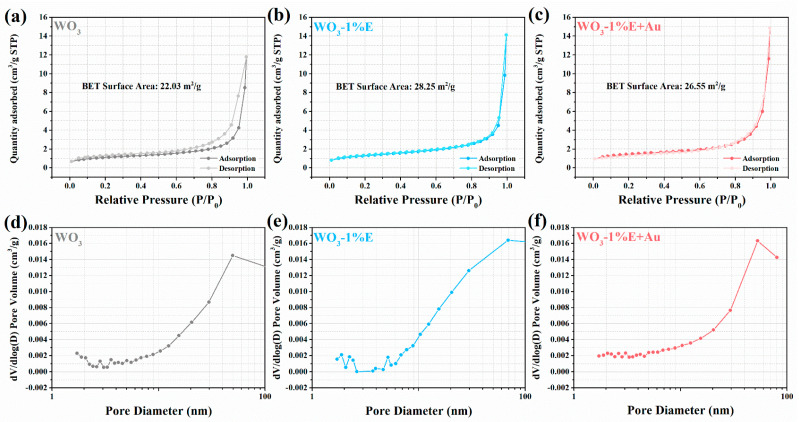
N_2_ adsorption-desorption isotherm and pore size curves of (**a**,**d**) WO_3_, (**b**,**e**) WO_3_-1%E, (**c**,**f**) WO_3_-1%E+Au.

The measured BET surface areas of WO_3_, WO_3_-1%E, and WO_3_-1%E+Au are 22.03, 28.25, and 26.55 m^2^/g, respectively (Figure 5a–c). All N_2_ adsorption-desorption isotherms showed typical IV isotherms with hysteresis loops in the high relative pressure range [44]. The pore size distribution curves of all samples separately are shown in Figure 5d–f. The BJH pore size distribution curves all peaked around 50 nm, indicating that both samples have microporous structures. These microporous structures are due to the stacking of WO_3_ particles, which is favorable for the transmission of the detected gas and contact with the sensing material.

### 3.2. Gas Sensing Performance

**Figure 6:**
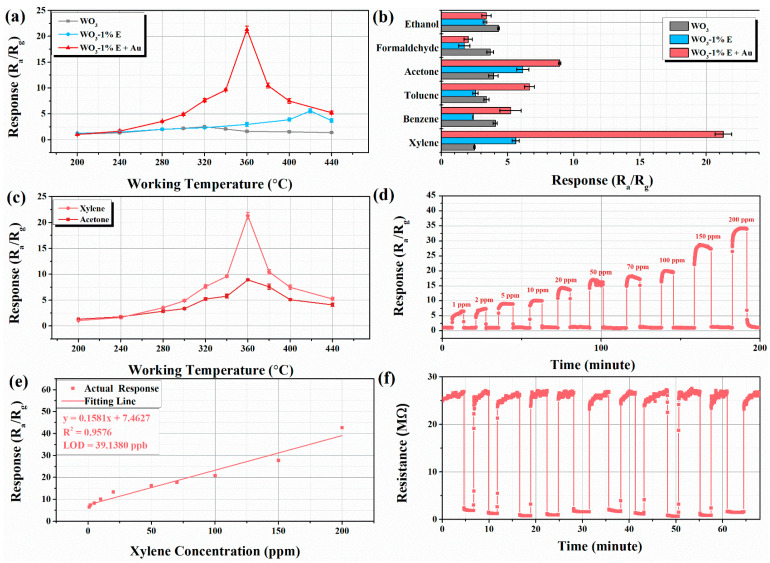
Response of all samples (**a**) to 100 ppm xylene at various temperatures, (**b**) to different 100 ppm gases at their optimal working temperature, and (**c**) to xylene and acetone at various temperatures. (**d**) Response curves for different concentrations of xylene at 360 °C. (**e**) Linear correlation between response and concentration at 360 °C. (**f**) Repeatability test of WO_3_-1%E+Au sample.

Figure 6a shows the response values of all sensors to xylene at different operating temperatures. All sensors showed a tendency to increase and then decrease as the operating temperature increased. Compared with other sensors, WO_3_-1%E+Au had the highest response value of 21.3 to 100 ppm xylene at the optimum working temperature of 360 °C. In addition, the gas-sensitive properties of WO_3_ etched with different concentrations of NaHCO_3_ and decorated with different amounts of Au to xylene were analyzed, as shown in Figures S5 and S6. The results showed that 1% NaHCO_3_ and 1% Au was the optimal ratio when the sensor obtained the best sensing performance to xylene.

The selectivity of the sensor is of great value in practical applications. Figure 6b shows the selectivity of the sensors towards 100 ppm of different target gases at 360 °C, including xylene, benzene, toluene, acetone, formaldehyde, and ethanol. In the figure, the response value of WO_3_-1%E+Au sensor for xylene is significantly higher than the other gases, which indicates its high selectivity towards xylene. It is noteworthy that WO_3_ and WO_3_-1%E exhibited a higher selectivity to acetone than the WO_3_-1%E+Au sensor. After the introduction of Au, the selectivity of the sensor to the gas changed, indicating that Au facilitates the response to xylene. Figure 6c presented that the WO_3_-1%E+Au sensor exhibited higher response values for xylene than for acetone over a wide temperature range of 280–440 °C, indicating the excellent selectivity of the WO_3_-1%E+Au sensor for xylene.

Figure 6d shows the dynamic sensor response of the WO_3_-1%E+Au sensor at 360 °C as a function of different xylene concentrations. As depicted, the response of the sensor increased with increasing gas concentration. At the end of each concentration detection, the device resistance value was fully recovered to the initial state after the removal of xylene. As shown in Figure 6d, xylene concentrations as low as 1 ppm can be detected with a response value of 6.48. A linear relationship (y = 0.1581x + 7.4627) was fitted based on the relationship between the sensor response value and the xylene concentration (Figure 6e). The correlation coefficient R^2^ of the fitted results is 0.9576, indicating a very good fit. According to the fitted curve, the theoretical limit of detection (LOD) [45,46] for the sensor can be calculated as 39.138 ppb.

The repeatability of the WO_3_-1%E+Au sensor was tested for 10 cycles at 360 °C, and the resistance signal curve is shown in Figure 6f. It can be seen that the fabricated sensor responds and recovers quickly to 100 ppm xylene with no significant fluctuation in the response resistance, which indicates that the sensor has good reproducibility.

**Figure 7:**
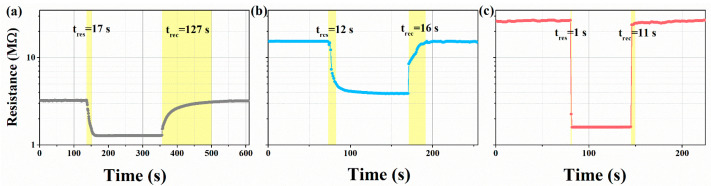
Dynamic response-recovery curves of (**a**) WO_3_, (**b**) WO_3_-1%E, (**c**) WO_3_-1%E+Au to 100 ppm xylene at their respective optimal working temperatures.

Considering the importance of the response and recovery speeds for the practical application of the sensor, the response/recovery time of the prepared sensors was examined as shown in Figure 7a–c. The response/recovery times were tested for 100 ppm xylene at their respective optimal operating temperatures: 17/127 s (WO_3_), 12/16 s (WO_3_-1%E), 1/11 s (WO_3_-1%E+Au). The above studies show that the NaHCO_3_ etchant-treated WO_3_ (namely, WO_3_-1%E and WO_3_-1%E+Au) sensitive materials have faster response/recovery. This is related to the uniform macroporous structure of the sensitive materials, which is favorable for the contact between the gas and the sensitive materials and the transport of the gas. The introduction of the Au element as a catalyst accelerated the reaction of xylene gas with the sensitive materials, resulting in the fastest response/recovery for WO_3_-1%E+Au.

**Figure 8:**
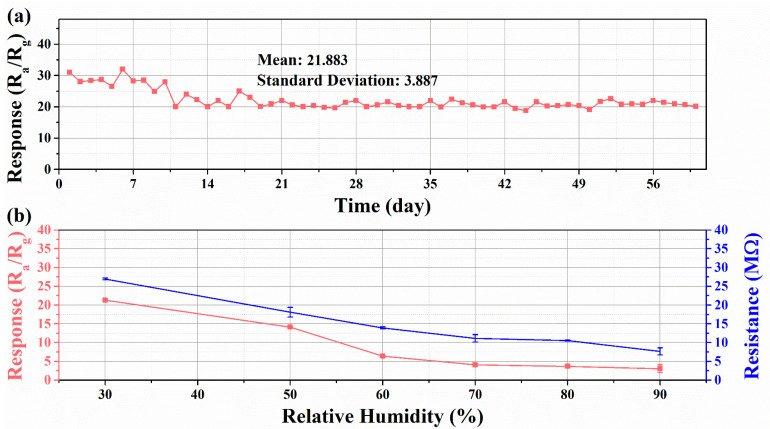
(**a**) Long-term stability test of WO_3_-1%E+Au at 360 °C and (**b**) response of WO_3_-1%E+Au to 100 ppm xylene at 360 °C and resistance change in air at various humidity.

The WO_3_-1%E+Au sensor was tested continuously for 60 days under laboratory conditions to figure out the long-term stability. The variation of the response value with time was plotted in Figure 8a. The response values of the sensor fluctuated slightly in the first 20 days but gradually stabilized over time. There is no significant decay in the response value during the measurement. The mean and standard deviation of the response values within 60 days were calculated to be 21.883 and 3.887, respectively, indicating that the sensor can be used for xylene detection in practical situations and the sensing performance remains stable [47].

The effect of humidity on sensor performance should not be ignored, as sensors are often exposed to different humidity environments in actual test scenarios [48]. Figure 8b shows the response and resistance changes of the WO_3_-1%E+Au sensor to 100 ppm xylene at various relative humidities. As depicted, the initial resistance and response value decreases to some extent as the relative humidity increases. The reason for this phenomenon is that water molecules react with adsorbed oxygen to form hydroxyl groups on the surface of the sensitive material and release electrons, so the resistance value of the sensor decreases in high-humidity environments [49,50]. In addition, water molecules have a competitive relationship with xylene gas [51], so the response value of the sensor deteriorates in high-humidity environments.

### 3.3. Sensing Mechanisms

**Figure 9:**
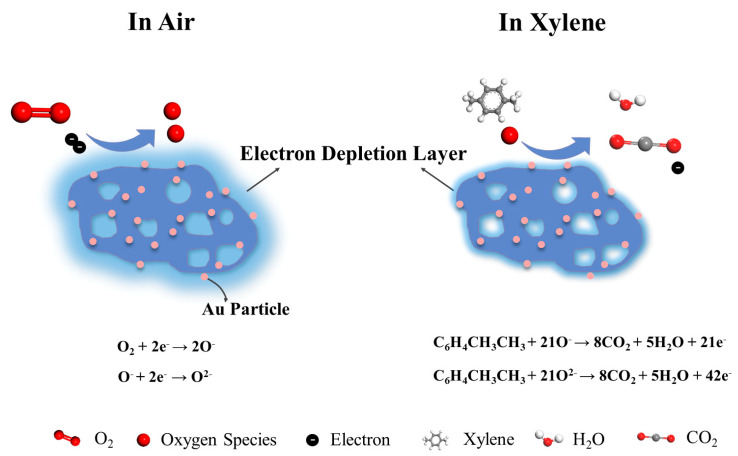
Schematic of the simulated reaction mechanism of WO_3_-1%E+Au with xylene.

As an n-type MOS, the main carriers in WO_3_ are electrons, and WO_3_ primarily relies on the surface interaction between the adsorbed oxygen and the target gas to generate electron transfer and cause resistance changes. The whole sensing and detection process involves a sequence of reactions of adsorption, oxidation, and desorption (Figure 9). When exposed to air, WO_3_ absorbs oxygen molecules from the air, and electrons in the WO_3_ conduction band will be captured by the absorbed oxygen to form oxygen species ions (O_2_^−^, O^−^ and O^2−^). The reaction process can be described using the following formulas [52]:

$$O_{2}\left(gas\right)\to O_{2}(ads)$$

$$O_{2}\left(ads\right)+e^{-}\to O_{2}^{-}\left(ads\right)(T<100\;{}^{\circ})$$

$$O_{2}^{-}\left(ads\right)+e^{-}\to 2O^{-}\left(ads\right)(100\;{}^{\circ}C<T<300\;{}^{\circ}C)$$

$$O^{-}\left(ads\right)+e^{-}\to O^{2-}\left(ads\right)(T>300\;{}^{\circ}C)$$

Upon exposure to a reducing gas such as xylene, the xylene molecules will interact with the oxygen species adsorbed on the surface and release the captured electrons back into the conduction band. This results in a decrease in the thickness of the electron depletion layer, an increase in conductivity, and a corresponding decrease in sensor resistance. The reaction between xylene and oxygen species can be expressed by the following formulas [53]:

$$C_{6}H_{4}CH_{3}CH_{3}\left(ads\right)+21O^{-}\left(ads\right)\to 8CO_{2}+{5H}_{2}O+21e^{-}$$

$$C_{6}H_{4}CH_{3}CH_{3}\left(ads\right)+21O^{2-}\left(ads\right)\to 8CO_{2}+{5H}_{2}O+42e^{-}$$

The enhanced gas sensing performance of WO_3_-1%E+Au could be attributed to the following mechanisms:

NaHCO_3_ etching causes defects on the WO_3_ surface, which increases the specific surface area and provides more active sites for gas adsorption. The increase in surface roughness and porosity also contributes to the improvement of gas diffusion and transport and increases the interaction between gas molecules and the sensing material. Moreover, the active sites generated by etching help to enhance the adsorption and catalytic properties [54]. This will promote the reaction between the target gas and the WO_3_ material, thus effectively enhancing the gas-sensing performance of the sensor.

The work function mismatch between Au (Φ = 5.1 eV) and WO_3_ (Φ = 4.4 eV) creates a potential barrier, causing majority carriers to accumulate at the interface between Au and WO_3_ [55,56]. The formation of a Schottky barrier increases the thickness of the electron depletion layer, providing more available carriers for oxygen adsorption in air [57]. Meanwhile, Au is able to promote the dissociation of oxygen molecules, resulting in highly reactive chemisorbed oxygen ions [26]. Additionally, Au acts as a catalyst to accelerate the surface reaction. This combined effect of enhanced oxygen dissociation and catalytic activity enables the sensor to exhibit excellent sensing characteristics [58].

The introduction of Au particles improves the selectivity for xylene. This is due to the fact that the strong electronic effect of xylene gas molecules can form a coupling effect with specific noble metals, which significantly affects the adsorption behavior of the gas molecules on metals [26]. Au nanoparticles catalyze the dehydrogenation reaction of C-H bonds in xylene [59]. Moreover, the planar structure of the benzene ring and Π conjugated system in xylene make it more reactive with oxygen ions adsorbed on the surface of the Au-loaded sensor than other VOC gases [60,61,62].

## 4. Conclusions

In this paper, Au was introduced together with NaHCO_3_ as an etchant to prepare WO_3_-1%E+Au with a homogeneous macroporous structure and rough surface. The carbon sphere template and the etchant worked in concert to create a lot of pores during this process, and the etched samples showed a higher response value and faster response/recovery rate than the pristine WO_3_. In addition, the Au decoration greatly enhanced the response of the WO_3_-1%E+Au sensor to xylene, and more importantly, the selectivity of the sensor to xylene was optimized. These findings highlight the importance of surface modification in enhancing gas sensor performance and are significant for improving the selectivity of volatile organic compounds (VOCs) gas sensors.

## Data Availability

No new data were created or analyzed in this study.

## Supplementary Materials

The following supporting information can be downloaded at: https://www.mdpi.com/article/10.3390/mi16060646/s1, Figure S1 SEM image of carbon spheres, Figure S2 Low magnification SEM image of WO_3_-1%E, Figure S3 SEM images of samples using different amount of NaHCO_3_ as etchant: (a) 0.5% NaHCO_3_, (b) 1% NaHCO_3_, (c) 3% NaHCO_3_ and (d) 5% NaHCO_3_, Figure S4 The XPS O1s spectra of (a) WO_3_, (b) WO_3_-1%E, Figure S5 The samples using different amount of NaHCO_3_ as etchant: (a) optimum working temperature to 100 ppm xylene, (b) responses to 100 ppm different target gases at 420 ℃, (c–f) dynamic response-recovery curves to 100 ppm xylene at 420 ℃, Figure S6 The WO_3_-1%E samples adding different molar ratio of Au atom: (a) optimum working temperature to 100 ppm xylene, (b) responses to 100 ppm different target gases at their optimum operating temperature, (c–f) dynamic response-recovery curves to 100 ppm xylene at their optimum operating temperature.
