# Supplementary material for: Etched Tungsten Oxide Modified with Au for Quick Xylene Detection
Source: Micromachines (Basel). 2025 May 28;16(6):646. doi: 10.3390/mi16060646 (PMC12195401; doi:10.3390/mi16060646)
Supplement: Supplementary file 1 [file micromachines-16-00646-s001.zip › micromachines-3664327-supplementary.pdf]

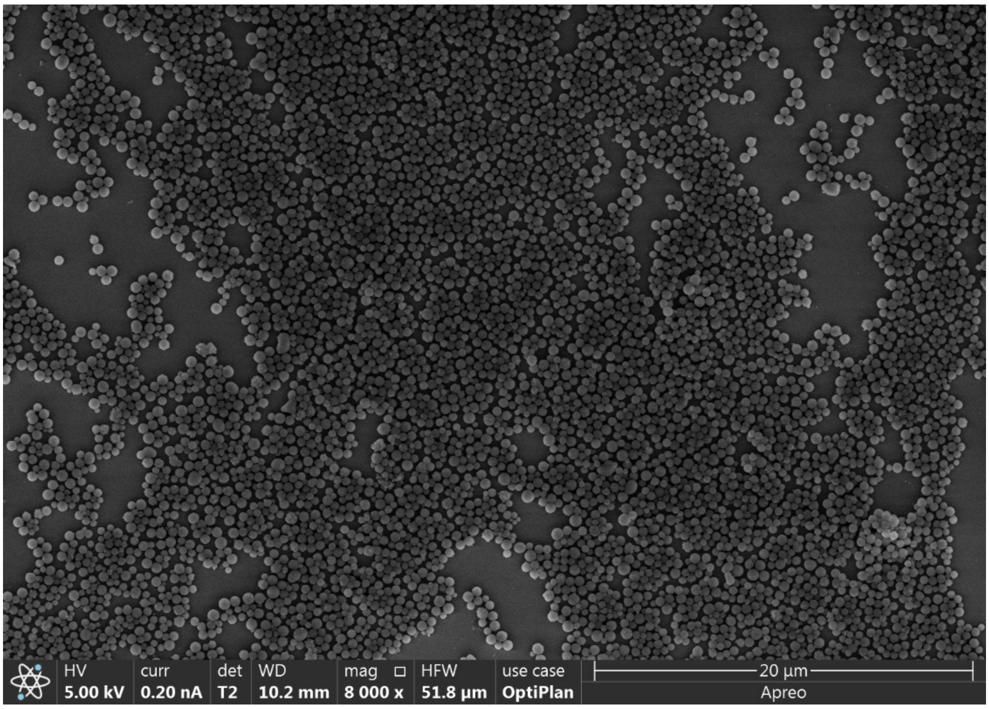

Fig. S1 SEM image of carbon spheres

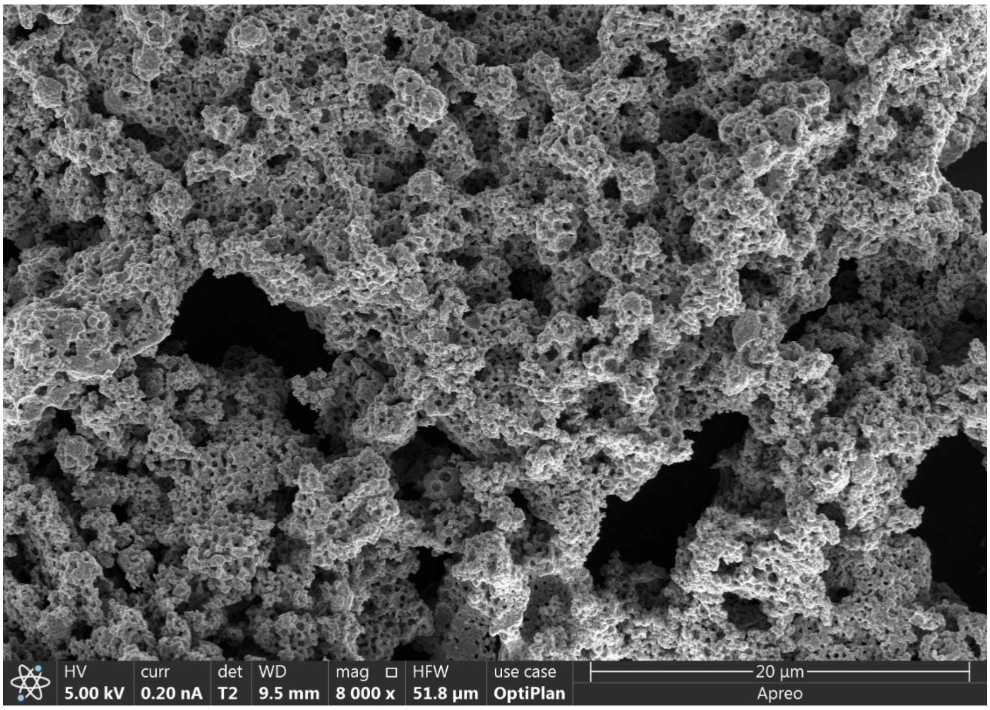

Fig. S2 Low magnification SEM image of WO_3-1%E

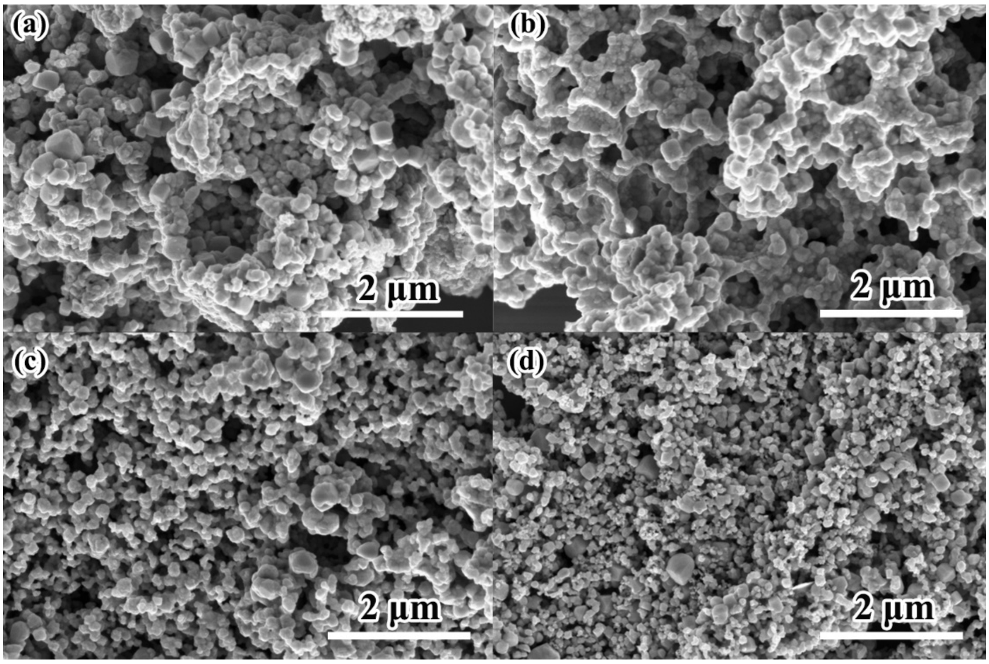

Fig. S3 SEM images of samples using different amount of $NaHCO_3$ as etchant: (a) 0.5% $NaHCO_3$, (b) 1% $NaHCO_3$, (c) 3% $NaHCO_3$ and (d) 5% $NaHCO_3$

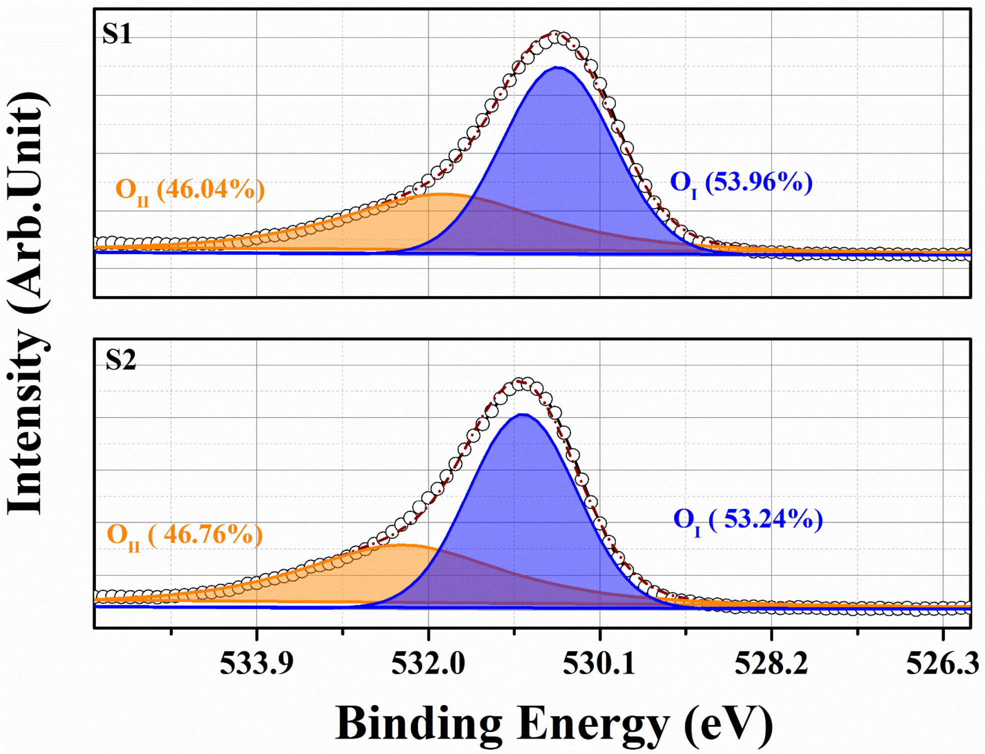

Fig. S4 The XPS O1s spectra of (a) WO_3, (b) WO_3-1%E.

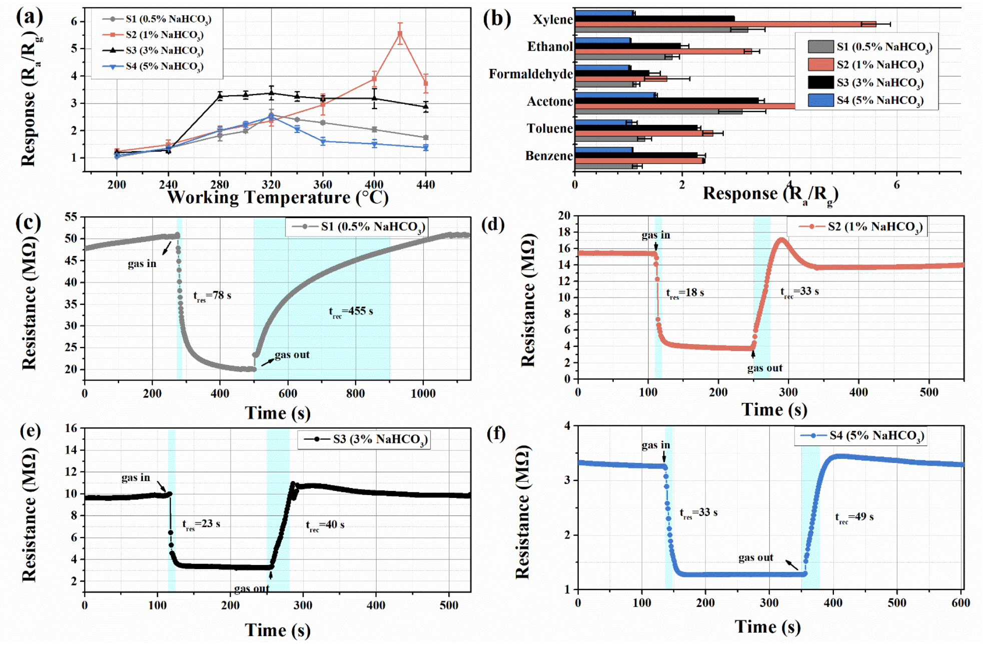

Fig. S5 The samples using different amount of NaHCO3 as etchant: (a) optimum working temperature to 100 ppm xylene, (b) responses to 100 ppm different target gases at 420 °C, (c)-(f) dynamic response-recovery curves to 100 ppm xylene at 420 °C.

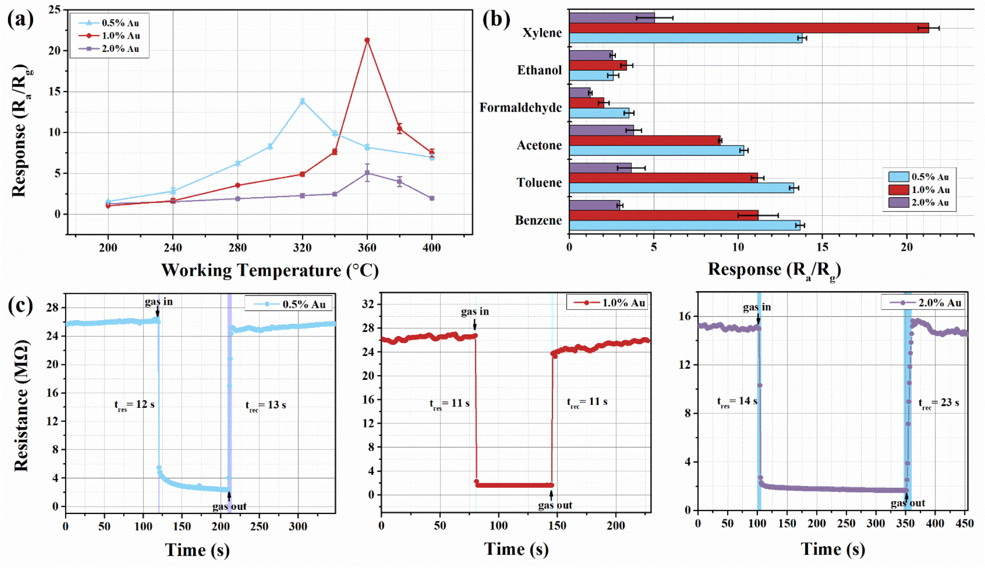

Fig. S6 The WO3-1%E samples adding different molar ratio of Au atom: (a) optimum working temperature to 100 ppm xylene, (b) responses to 100 ppm different target gases at their optimum operating temperature, (c)-(f) dynamic response-recovery curves to 100 ppm xylene at their optimum operating temperature.
